# Intermittent screening and treatment for malaria complementary to routine immunisation in the first year of life in Papua, Indonesia: a cluster randomised superiority trial

**DOI:** 10.1186/s12916-022-02394-1

**Published:** 2022-06-08

**Authors:** Jeanne Rini Poespoprodjo, Hafiidhaturrahmah, Novita Sariyanti, Ratni Indrawanti, Alistair R. D. McLean, Julie A. Simpson, Enny Kenangalem, Faustina Helena Burdam, Rintis Noviyanti, Leily Trianty, Chairunisa Fadhilah, Yati Soenarto, Ric N. Price

**Affiliations:** 1 Centre for Child Health and Department of Child Health, Faculty of Medicine, Public Health and Nursing, Universitas Gadjah Mada, Jl. Kesehatan no.1, Sekip, Yogyakarta, 55284 Indonesia; 2 Timika Malaria Research Facility, Papuan Health and Community Development Foundation, Jl. SP2-SP5, RSMM Area, Timika, Papua 99910 Indonesia; 3 Mimika District Hospital and District Health Authority, Jl. Yos Sudarso, Timika, Papua 99910 Indonesia; 4 Centre for Epidemiology and Biostatistics, Melbourne School of Population and Global Health, University of Melbourne, 207 Bouverie Street, VIC 3010 Melbourne, Australia; 5 Eijkman Institute for Molecular Biology, Jl. Diponegoro No.69, Jakarta, 10430 Indonesia; 6 Global Health Division, Menzies School of Health Research and Charles Darwin University, PO Box 41096, Casuarina, Darwin, NT 0811 Australia; 7 Centre for Tropical Medicine, Nuffield Department of Clinical Medicine, University of Oxford, Oxford, OX37LJ UK; 8 Mahidol-Oxford Tropical Medicine Research Unit (MORU), Faculty of Tropical Medicine, Mahidol University, Bangkok, Thailand

**Keywords:** Intermittent screening and treatment, Infant, Malaria, High malaria transmission, Asia Pacific

## Abstract

**Background:**

In Papua (Indonesia), infants with *P. falciparum* and/or *P. vivax* malaria are at risk of severe anaemia and death. We hypothesized that in an area of high malaria transmission, intermittent screening and treatment of infants with malaria (ISTi) will reduce morbidity compared to passive case detection (PCDi).

**Methods:**

We conducted a cluster randomised, open label, superiority trial. A total of 21 clusters of village health posts (VHP) were randomised 1:1 to either IST for infants coinciding with 4 routine immunisation visits or PCDi. Healthy term infants born to consenting mothers enrolled into a maternal malaria cluster randomised trial were included in the study and followed for 12 months. Point of care malaria rapid diagnostic tests were used to detect peripheral parasitaemia at 2, 3, 4 and 9 months old in all infants in ISTi clusters and when symptomatic in PCDi clusters. Infants with detected peripheral parasitaemia were treated with dihydroartemisinin-piperaquine. The co-primary outcomes were the incidence rate of clinical malaria in the first year of life and the prevalence of parasitaemia at age 12 months. The incidence rate ratio and prevalence ratio between ISTi and PCDi were estimated using mixed-effects Poisson and log-binomial regression modelling (accounting for clustering at VHP level).

**Results:**

Between May 2014 and February 2017, 757 infants were enrolled into the study, 313 into 10 ISTi clusters, and 444 into 11 PCDi clusters. Overall, 132 episodes of parasitaemia were detected, of whom 17 (12.9%) were in symptomatic infants. Over 12 months, the incidence rate (IR) of clinical malaria was 24 [95% CI, 10–50] per 1000 children-years at risk in the ISTi arm and 19 [95% CI, 8,38] per 1000 children-years in the PCDi arm (adjusted incidence rate ratio [aIRR] 1.77 [95% CI, 0.62–5.01]; *p* = 0.280). The prevalence of parasitaemia at 12 months was 13% (33/254) in the IST clusters and 15% (57/379) in the PCD clusters (adjusted prevalence ratio (aPR) = 0.92 (95% CI, 0.70–1.21), *p* = 0.55). There was no difference in the risk of anaemia between treatment arms.

**Conclusions:**

In high malaria transmission area outside of Africa, our study suggests that compared to PCDi, ISTi offers no significant benefit in reducing the risk of clinical malaria in infants born to women receiving effective protection from malaria during pregnancy.

**Trial registration:**

ClinicalTrials.gov NCT 02001428, registered on 20 Nov 2013.

**Supplementary Information:**

The online version contains supplementary material available at 10.1186/s12916-022-02394-1.

## Background

In malaria endemic areas, the susceptibility of infants to malaria increases as maternal antibodies wane after birth, rendering infants at risk of severe disease and dying [1–4]. In Timika, Papua (Indonesia), *Plasmodium falciparum* and *P. vivax* are highly prevalent and a third of hospitalised infants are diagnosed with malaria, often associated with severe anaemia and respiratory distress in the first year of life [2]. Infants and children under-5 years of age are also more prone to repeated episodes of *P. vivax* malaria than older children and adults. If undetected and inadequately treated, recurrent parasitaemia leads to a cumulative risk of severe anaemia and death [5–7].

In infancy, the signs and symptoms of malaria are not specific, leading to a delay in diagnosis and treatment [2]. Early diagnosis and prompt effective treatment can prevent the adverse outcomes [8] but is logistically challenging to implement, particularly in places where resources are limited and access to healthcare is poor.

In areas of high transmission of *P. falciparum* in Africa, the implementation of intermittent preventive treatment in infancy (IPTi) with curative dose of sulfadoxine-pyrimethamine (SP) has been shown to be an effective malaria control strategy [9, 10]. However, in co-endemic regions in Asia and the Pacific, the evidence of benefit is weaker with only one study demonstrating the effectiveness of IPTi with SP and amodiaquine in reducing malaria in the first year of life [11]. In areas where SP resistance is high, monthly dihydroartemisinin-piperaquine (DHP) has been proposed as an alternative regimen for IPTi [12].

DHP has been the first-line treatment for uncomplicated malaria in Indonesia since 2006, and its use as monthly IPT during pregnancy has been shown to be effective in reducing the risk of maternal malaria [13]. However, its applicability to infants in such settings requires additional evidence on its feasibility and acceptability [14]. Due to concerns of administering antimalarial drugs without testing in infants and pregnant women living in an area of high malaria transmission, intermittent screening and treatment (IST) may provide an alternative strategy to IPT [13, 15–17]. However, to date, no studies have examined the effectiveness of intermittent screening and treatment in the first year of life. We undertook a cluster randomised controlled trial to evaluate whether ISTi was more effective than passive case detection (PCDi) in reducing the incidence of clinical malaria in the first year of life and prevalence of parasitaemia at 12 months of age infants living in an area of high malaria transmission.

## Methods

### Trial design

Between May 2014 to March 2017, we conducted a cluster randomised, open-label superiority trial to compare ISTi (intervention group) with PCDi (control group). Intermittent screening coincided with the infant’s local immunisation schedule. The unit of randomisation was the village health post (VHP), a community-based maternal and child health care facility, providing monthly antenatal care for pregnant women, as well as an immunisation and nutritional program for infants and children under 5 years old. A total of 21 VHPs were randomised to implement either ISTi or PCDi.

### Participants

Healthy term infants born from mothers participating in a maternal cluster randomised trial were eligible to be enrolled into the infant study. The maternal study compared three arms: intermittent screening and treatment (ISTp), intermittent preventive treatment (IPTp) and single screening and treatment (SSTp), as described previously [13]. Infants were enrolled at birth into the complementary infant study and followed from 6 weeks old until 12 months. Written informed consent was obtained from the parents at birth.

### Study area

The study was conducted in Timika, Papua (Indonesia), an area where *P. falciparum* and *P. vivax* parasitaemia are similarly prevalent. Previous clinical trials in this region have shown high rates of treatment failure for patients with either *P. falciparum* or *P. vivax* malaria following chloroquine monotherapy, CQ plus SP or unsupervised quinine [18]. In 2006, antimalarial treatment guidelines were revised to recommend DHA-piperaquine as first treatment for uncomplicated malaria due to all Plasmodium species. The Annual Parasite Incidence (API) at the start of the study was 224 per 1000 person years but rose to 432 per 1000 person years when the study ended in 2017, divided equally between *P. falciparum* and *P. vivax* infections [19].

### Randomisation and blinding

The 21 clusters were stratified by the three maternal intervention groups (7 per group) as described elsewhere [13]. Within each maternal intervention group, the clusters were paired according to malaria transmission and then for each pair of clusters one was randomly allocated to ISTi and one to PCDi, and the one that could not be paired randomly allocated to either ISTi or PCDi. The randomisation was carried out by an independent statistician using Stata version 15.1 (StataCorp, College Station, TX USA).

Study participants, field team, investigators and study statistician were aware of the intervention allocation, however laboratory technicians responsible for deriving the clinical outcomes were blinded to intervention allocation.

### Procedures

At birth, information on maternal and newborn’s demographic, socioeconomic, clinical and laboratory data were collected from mother and child records reported in the maternal study. After 6 weeks of age, clinical and laboratory data were collected prospectively by the investigators of the infant study.

All infants were followed up until 12 months of age. Infants in all clusters underwent a routine clinical questionnaire and examination (including anthropometric data) at 2, 3, 4, 6, 9 and 12 months old. The diagnosis of malaria and asymptomatic parasitaemia was made at the VHP using rapid diagnostic tests (SD Bioline malaria Ag Pf/Pan-Inc-05FK60), with microscopic peripheral blood examination conducted later the same day at the supporting study laboratory. Slides were read by two experienced microscopists. In cases where readings were discordant, the slides were reread by a third microscopist and a consensus reached. Haemoglobin (Hb) concentration was measured using a portable haematocrit analyser (Hemocue®).

In the ISTi clusters, infants were screened routinely for peripheral parasitaemia and had a Hb measurement at 2, 3, 4 and 9 months of age coinciding with diphtheria, pertussis, tetanus (DPT) 1-2-3 and measles vaccination, respectively. In the PCDi group, infants were only assessed for peripheral parasitaemia and Hb if they presented with fever or history of fever in the preceding 24 h. Additional capillary blood samples were collected during home visits from a heel prick from all infants in ISTi and PCDi clusters at age 6 and 12 months of age. Submicroscopic infections and speciation were assessed at the 12-month visit using qPCR methods to blood samples collected, as described previously [20, 21].

Infants with clinical malaria in either group and those with asymptomatic peripheral parasitaemia in the ISTi arm were treated with DHP once daily for 3 days (total dose of 6 mg/kg body weight of dihydroartemisinin and 57 mg/kg body weight of piperaquine) without primaquine according to the national treatment guidelines [22]. Individuals with peripheral parasitaemia diagnosed by microscopy but not initially by RDT were visited by a field worker on the following day to administer similar antimalarial treatment. Infants with signs of severe malaria were reviewed by the research clinician and referred to the local hospital if necessary. All severe adverse events were documented and reviewed by the Data and Safety Monitoring Board (DSMB) (Additional file 1: p 8). The families of all infants were provided with a long lasting insecticide treated net (LLIN) at the start of maternal and infants’ trial.

### Outcomes

Clinical malaria was defined as the presence of fever (axillary temp ≥ 37.5^0^C) or history of fever in the preceding 24 h plus detection of peripheral asexual parasitaemia by either RDT or microscopy. The co-primary study outcomes were the incidence rate of clinical malaria in the first 12 months of life and the prevalence of peripheral parasitaemia (detected by either microscopy, RDT or quantitative polymerase chain reaction/qPCR) at 12 months of age regardless of symptoms. Secondary outcomes included the prevalence of anaemia (Hb < 10 g/dl) at 6 and 12 months.

### Statistical analysis

The 21 clusters were predetermined by the maternal cluster randomised controlled trial (RCT). With 757 infants, the trial was powered to detect a 20% reduction in the incidence rate of clinical malaria from 3.0 to 2.4 case per child in the first year of life [2] with 80% power at the 5% significance level, assuming 10% lost to follow-up and a conservative intra-cluster correlation coefficient (ICC) of 0.05. The latter was a conservative estimate, derived from the reported ICC of 0.018 in a cluster RCT of intermittent preventive treatment in children in Senegal [23].

Data were double entered and validated using EpiData 3.02 software (EpiData Association, Odense, Denmark) and analysis performed using Stata version 17.0. Demographic data were presented by intervention arm with categorical variables presented as frequency (%) and continuous variables as mean (SD) or median (25th-75th percentiles). The adjusted incidence rate ratio (IRR, 95% confidence interval (CI)) of clinical malaria in the first year of life comparing IST to PCD intervention group was estimated using mixed-effects Poisson regression analysis, with the exposure period calculated using the age at final visit, adjusted for maternal intervention group, maternal parasitaemia at enrolment or during pregnancy, maternal socioeconomic status (low/medium/high) and infant sex. Random effects at the VHP level were used to account for clustering. Clinical assessments of the 12-month visit that occurred after 14 months (425 days) were censored in the analysis.

The adjusted prevalence ratio (PR, 95% CI) of parasitaemia at age 12 months was estimated using log binomial regression analysis with a robust estimator of standard error adjusted for maternal intervention group, maternal parasitaemia at enrolment or during pregnancy, maternal socioeconomic status (low/medium/high) and infant sex. The adjusted prevalence ratio (aPR, 95% CI) of anaemia at age 6 and 12 months was estimated using a log binomial regression analysis with a robust estimator of standard error adjusted for maternal intervention group, maternal parasitaemia at enrolment or during pregnancy, maternal socioeconomic status, maternal anaemia (Hb < 10 g/dl), maternal malnutrition (mid upper arm circumference < 23.5 cm) and infant’s sex. The modified intention to treat population included all randomised participants with outcome data available.

Ethical approval was obtained from the Human Research Ethics Committee of the Northern Territory Department of Health, Australia (HREC 2013-2108), and the Health Research Ethics Committees of the Faculty of Medicine Universitas Gadjah Mada, Indonesia (KE/FK/1023/EC/2013).

## Results

**Fig. 1:**
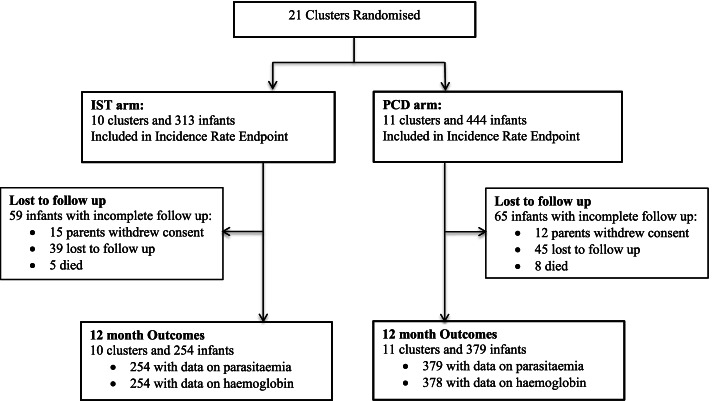
Study profile

The study was conducted over 33 months with the first recruitment on 7 May 2014, the last patient enrolled on 6 April 2016 and the last follow-up visit on 23 February 2017. A total of 757 infants (755 singletons and 1 set of twins) were enrolled into the study (Fig. 1). Infant characteristics are presented in Table 1. A greater number of infants in ISTi arms were from maternal IST group (47.1%) compared to those in PCDi arms (32.7%) (Table 2), whereas more infants in PCDi arms were from maternal IPTp-DHP group compared to those in ISTi arms (35.4% and 22.0% respectively). In addition, higher proportion of infants in PCDi arms had mothers with anaemia and severe anaemia (25.6% and 3.2% respectively) compared to those in ISTi arms (18.6% and 1% respectively). At delivery, 18.6% (128/686) of women had parasitaemia (peripheral or placental) with a prevalence of maternal parasitaemia of 17.5% (50/285) in the ISTi arm and 19.5% (78/401) in the PCDi arms (Additional file 1: p 14).

**Table 1:** Infant characteristics overall and by infants’ intervention groups

	ISTi (10 clusters)	PCDi (11 clusters)	Total
	*N* = 313	*N* = 444	*N* = 757
Birthweight (grams)^a^	3045 (2780, 3330)	3020 (2770, 3320)	3030 (2770, 3320)
Low birth weight (< 2500 g)^b^	29/286 (10%)	37/401 (9.2%)	66/687 (9.6%)
Birthweight > 3500 g^b^	40/286 (14%)	55/401 (14%)	95/687 (14%)
Infant sex (female)^b^	147/313 (47%)	219/444 (49%)	366/757 (48%)
**Foetal Hb and anaemia**			
Foetal (cord) Hb (g/dL)^a^	14.7 (3.3)	14.7 (3.0)	14.7 (3.2)
Foetal (cord) Hb < 10 g/dL^b^	24/271 (8.9%)	26/381 (6.8%)	50/652 (7.7%)
Foetal (cord) Hb < 7 g/dL^b^	7/271 (2.6%)	3/381 (0.8%)	10/652 (1.5%)
**Cord malaria by microscopy**			
*P. falciparum*^b^	1/211 (0.5%)	1/322 (0.3%)	2/533 (0.4%)
*P. vivax*^b^	0/211 (0.0%)	0/322 (0.0%)	0/533 (0.0%)
**Cord malaria by PCR**			
*P. falciparum*^b^	2/272 (0.7%)	10/381 (2.6%)	12/653 (1.8%)
*P. vivax*^b^	2/272 (0.7%)	10/381 (2.6%)	12/653 (1.8%)
**Cord malaria (microscopy or PCR)**^b^	4/272 (1.5%)	13/381 (3.4%)	17/653 (2.6%)
*P. falciparum*^b^	3/273 (1.1%)	11/381 (2.9%)	14/654 (2.1%)
*P. vivax*^b^	2/273 (0.7%)	10/381 (2.6%)	12/654 (1.8%)

*ISTi* intermittent screening and treatment in infants, *PCDii* passive case detection in infants

^a^Continuous data are presented as median (25^th^ percentile, 75^th^ percentile) or mean (SD)

^b^Categorical data are presented as frequency in valid cases (%)

**Table 2:** Maternal characteristics of infants enrolled into the study

	ISTi (10 clusters)	PCDi (11 clusters)	Total
	***N*** = 313	***N*** = 444	***N*** = 757
Age (years)^b^	26 (6)	25 (6)	25 (6)
Parity^b^	2 (1, 3)	2 (1, 3)	2 (1, 3)
**Parity group**^a^			
Primiparity	85/311 (27.3)	133/443 (30.0)	218/754 (28.9)
Multiparity	226/311 (72.7)	310/443 (69.9)	536/754 (71.1)
**Socioeconomic status**^a^			
Low	93/313 (29.7)	177/444 (39.9)	270/757 (35.7)
Medium	103/313 (32.9)	133/444 (29.9)	236/757 (31.2)
High	117/313 (37.4)	134/444 (30.2)	251/757 (33.2)
**Education**^a^			
No school	37/294 (12.6)	44/407 (10.8)	81/701 (11.6)
Primary and secondary school	109/294 (37.1)	203/407 (49.9)	312/701 (44.5)
High School	14/294 (4.8)	18/407 (4.4)	32/701 (4.6)
College education	134/294 (45.6)	142/407 (34.9)	276/701 (39.4)
**Maternal intervention groups**^ac^			
SST	97/313 (31.1)	142/444 (32.1)	239/757 (31.6)
IST	147/313 (47.1)	145/444 (32.7)	292/757 (38.6)
IPT-DHP	69/313 (22.0)	157/444 (35.4)	226/757 (29.9)
**Any maternal malaria at antenatal care (any method)**^a^	89/313 (28.4)	132/444 (29.7)	221/757 (29.2)
*P. falciparum*	61/313 (19.5)	92/444 (20.7)	153/757 (20.2)
*P. vivax*	57/313 (18.2)	92/444 (20.7)	149/757 (19.7)
**Peripheral parasitaemia (any method) at delivery**^a^	29/284 (10.2)	46/400 (11.5)	75/684 (11.1)
*P. falciparum*	16/283 (5.7)	30/400 (7.5)	46/683 (6.7)
*P. vivax*	18/284 (6.3)	35/399 (8.8)	53/683 (7.8)
**Any placental malaria infection at delivery**^a^	32/274 (11.7)	50/381 (13.1)	82/655 (12.5)
*P. falciparum*	11/282 (3.9)	22/399 (5.5)	33/681 (4.8)
*P. vivax*	16/283 (5.7)	31/401 (7.7)	47/684 (6.9)
**Any placental or peripheral malaria infection at delivery**	50/285 (17.5)	78/401 (19.5)	128/686 (18.7)
**Hb and anaemia at enrolment**			
Mean Hb (g/dL)^b^	11.4 (1.8)	11.0 (1.9)	11.2 (1.9)
Anaemia (Hb < 10 g/dL)^a^	58/311 (18.6)	113/442 (25.6)	171/753 (22.7)
Anaemia (Hb < 7 g/dL)^a^	3/311 (1.0)	14/442 (3.2)	17/753 (2.3)
**Hb and anaemia at delivery**			
Mean Hb (g/dL)^b^	11.3 (1.9)	11.2 (2.2)	11.2 (2.1)
Anaemia (Hb < 10 g/dL)^a^	54/261 (20.7)	99/367 (27.1)	153/628 (24.4)
Anaemia (Hb < 7 g/dL)^a^	2/284 (0.7)	16/400 (4.0)	18/684 (2.6)
**Maternal malnutrition**^a^	69/312 (22.1)	90/443 (20.3)	159/755 (21.1)

*ISTii* intermittent screening and treatment in infants, *PCDii* passive case detection in infants

^a^Categorical data are presented as frequency in valid cases (%)

^b^Continuous data are presented as median (25th percentile, 75th percentile) or mean (SD)

^c^*SSTi* single screening and treatment, *ISTi* intermittent screening and treatment, *IPT-DHPi* intermittent preventive and treatment with dihydroartemisinin-piperaquine

During the first 12 months of life, the total incidence of peripheral parasitaemia was 132 episodes of which 17 (12.8%) were in symptomatic infants. At 6 months, 1.0% (7/668) of infants had peripheral patent parasitaemia detected by RDT or microscopy (Additional file 1: p 2 and Table 3). At 12 months, peripheral parasitaemia, detected by any method, was present in 14.2% (90/633) of infants, of whom 88.9% (80/90) were asymptomatic and 78.9% (71/90) submicroscopic (qPCR positive but negative by microscopy or RDT); Table 3. There were no significant differences in the distributions of species of infection between the intervention arm. The geometric mean parasite density in infants with microscopically detected parasitaemia ranged from 13.1 to 94.2 parasites/μL for *P. falciparum* and from 34.2 to 85.0 parasites/μL for *P. vivax*.

**Table 3:** Prevalence of peripheral parasitaemia in infants at 6 and 12 months of age

Methods of detection	ISTi (10 clusters)	PCDi (11 clusters)	Total
	*N* = 313	*N* = 444	*N* = 757
**6 months**			
**By RDT**^a^			
*P. falciparum*	2/278 (0.7)	5/390 (1.3)	7/668 (1.0)
*P. vivax*	0/278 (0)	0/390 (0)	0/668 (0)
**By microscopy**			
*P. falciparum*	1/278 (0.4)	4/390 (1.0)	5/668 (0.7)
*P. vivax*	1/278 (0.4)	1/390 (0.3)	2/668 (0.3)
**12 months**			
**By RDT**			
*P. falciparum*	8/253 (3.2)	10/374 (2.7)	18/627 (2.9)
*P. vivax*	0/253 (0)	0/374 (0)	0/627 (0)
**By microscopy**			
*P. falciparum*	3/254 (1.2)	3/376 (0.8)	6/630 (1.0)
*P. vivax*	4/254 (1.6)	5/376 (1.3)	9/630 (1.4)
**By PCR**			
*P. falciparum* or mixed infections	21/247 (8.5)	40/362 (11.0)	61/609 (10.0)
*P. vivax* or mixed infections	6/247 (2.4)	13/362 (3.6)	19/609 (3.1)
**Sub-patent infections**			
*P. falciparum*	19/246 (7.7)	38/356 (10.7)	57/602 (9.5)
*P. vivax*	4/246 (1.6)	10/356 (2.8)	14/602 (2.3)
**Composite of any methods**	33/254 (13.0)	57/379 (15.0)	90/633 (14.2)

The total number of reported infections during follow-up was 132

*ISTii* intermittent screening and treatment in infants, *PCDii* passive case detection in infants

^a^PCR only performed in infants with malaria positive at 12 months. All values are presented as frequency in valid cases (%)

### Primary outcomes

At the end of follow-up the incidence rate (IR) of clinical malaria in the ISTi arm was 7 per 287 children years at risk (IR = 24 [95% CI, 10–50] per 1000 children-years) compared to 8 per 417 children-years at risk in the PCDi group (IR = 19 [95% CI, 8–38] per 1000 children-years); adjusted incidence rate ratio (aIRR) = 1.77 (95% CI, 0.62–5.01), *p* = 0.28. The prevalence of parasitaemia detected by any method (microscopy, RDT or qPCR) at 12 months was 13% (33/254) in the IST clusters and 15% (57/379) in the PCD clusters; adjusted prevalence ratio (aPR) = 0.92 (95% CI, 0.70–1.21), *p* = 0.55), Table 4.

**Table 4:** Infant parasitaemia and anaemia (Hb < 10 g/dl) at 6 and 12 months follow-up

Outcome	ISTi (***n***/***N*** (%))	PCDi (***n***/***N*** (%))	aPR (95% CI); ***p*** value
Any parasitaemia at 12 months	33/254 (13.1)	57/379 (15.0)	0.92 (0.70–1.21); 0.550
Anaemia at 6 months	66/278 (23.7)	112/392 (28.6)	0.86 (0.66–1.13); 0.280
Anaemia at 12 months	70/254 (28.1)	114/378 (30.2)	0.98 (0.81–1.18); 0.810

Composite of RDT, microscopy or PCR

Adjusted for maternal intervention group; maternal parasitaemia at enrolment or during pregnancy; maternal socioeconomic status (low/medium/high) and infant sex, with random effects at the VHP level to account for clustering

*aPR* adjusted prevalence ratio, *95% CI* 95% confidence interval, *ISTii* intermittent screening and treatment in infants, *PCDii* passive case detection in infants

### Secondary outcomes

Anaemia (Hb < 10 g/dl) was present in 26.7% (178/670) of infants at 6 months and 29.1% (184/632) at 12 months (Table 4). Overall 41% (37/90) infants with any parasitaemia at 12 months were anaemic compared to 27% (147/542) of those without any parasitaemia; *p* = 0.012. The prevalence of anaemia (Hb < 10 g/dl) was 24% (66/278) in the ISTi arm and 29% (112/392) in the PCD arm at 6 months (aPR = 0.86 (95%CI, 0.66-1.13), *p* = 0.280) 28% (70/254) and 30% (114/378) respectively at 12 months (aPR = 0.98 (95% CI, 0.81–1.18), *p* = 0.810); Table 4. Severe anaemia (Hb < 7 g/dl) was present in 1.6% (4/254) of infants aged 12 months old in the ISTi arm and 1.1% (4/378) in PCDi arm; *p* = 0.820.

### Severe adverse events

In total, 56 severe adverse events were reported. Four infants were hospitalised with severe malaria, two of whom were infants with vivax malaria and severe anaemia (1 in each treatment arm), one infant with falciparum malaria and hyperpyrexia (PCDi arm) and one with malaria due to mixed species infection (PCDi arm). All infants were treated with intravenous artesunate and DHP and made a full recovery (Additional file 1: p 9). One hospitalised infant with severe vivax malaria had malaria before enrolment into the study and her mother took her to a private clinic where she was treated with DHP. None of the cases of severe malaria were associated with the study intervention and all infants had cleared their parasitaemia by the next follow-up visit.

By 12 months of age, 39 infants were admitted to the hospital without malaria, with a similar risk of admission in both treatment arms: 6.4% (20/313) in the ISTi arm and 4.3% (19/444) in the PCDi arm. One infant in each arm required 2 admissions (Additional file 1: p 10). In total, 13 infants died (5 in the ISTi arm and 8 in the PCDi arm), with none of the deaths considered to be related to the study intervention. One death occurred at home since the parents refused medical attention and the rest died at the hospital (Additional file 1: page 8). The cause of death included tuberculous meningitis, severe congenital heart disease and severe sepsis. Overall, wasting (weight for height *z* score < -2SD and > − 3SD) was detected in 11% (74/678) of infants at 6 months and 15% (98/634) at 12 months with similar prevalence between intervention arms.

## Discussion

Our study highlights that in this area of high malaria transmission, the incidence of clinical malaria in the first year of life, in infants born of mothers offered protection from malaria during pregnancy, was low (incidence rate of 24 and 19 episodes per 1000 children-years for ISTi and PCDi respectively). Although there were a higher number of clinical malaria episodes in the ISTi clusters, the overall benefit of ISTi compared to PCDi arms in reducing clinical malaria was inconclusive. Overall 12.9% of the 132 detected episodes of parasitaemia were associated with symptomatic illness. By 12 months, 14% of infants had detectable peripheral parasitaemia, but almost 80% of these infections were submicroscopic.

The annual parasite incidence in the study area is approximately 250–400 cases per 1000 population, and thus the incidence of malaria in the infants enrolled was significantly lower than expected. Malaria in early life can result from vertical transmission (that may manifest months after birth), development of immune tolerance to malaria antigen in utero (increased susceptibility to malaria) or sporozoite inoculation by infected mosquitos in early life [24–27]. The low number of infected infants in our study may reflect the low (4%) prevalence of microscopic parasitaemia at delivery in mothers who had been carefully monitored during pregnancy with greater awareness in the need to protect their infants from malaria. Mothers participating in the maternal intervention study were also provided with LLIN. Although further infant studies could help clarify the potential benefits of ISTi in high risk children independent of any maternal interventions, a combined approach is warranted to improve maternal-infant health and the key question to address is the degree to which resources are focused pre or post natal.

Despite the low risk of infant malaria, almost 30% of participants were anaemic (Hb < 10 g/dl) by the time they were 12 months old. Although greater number of infants in PCDi arms had mothers with anaemia and severe anaemia, the risk of anaemia was similar between infants in ISTi and PCDi arms at 6 months (23.7% and 28.6% respectively, *p* = 0.280) and at 12 months (28.1% and 30.2% respectively, *p* = 0.810) suggesting minimal effect of maternal anaemia status in these infants. As well as malaria, nutritional deficiencies including iron are major causes of anaemia in early life [28] and associated with long-term developmental and cognitive impairment [29]. Anaemia in infancy occurs physiologically 8-12 weeks after birth with Hb falling to 9 g/dl, before increasing to 11–12 g/dl by 12–24 months of age providing that there are no other contributory causes of anaemia [30].

Maternal anaemia status was not considered a priori to be a confounding factor for the primary outcome. In a sensitivity analysis in which maternal anaemia was included as a covariate in the analysis; the estimated adjusted incidence rate ratio was similar to the findings of the primary analysis (aIRR of 1.79 [95% CI, 0.63-5.08] *p* = 0.274).

Among paediatric populations, test and treat strategies have been shown to have benefit in reducing malaria infections in school children and have been suggested as an alternative to IPT in moderate to highly malaria endemic areas [17]. Our study suggests that in an area with a high incidence of malaria, such a strategy during infancy offered no apparent benefit in reducing malaria. A key factor undermining ISTi in our study was that the very low level of peripheral parasitaemia, with 80% of detected cases being below the level of microscopic detection and in those with microscopic detectable infection, 95% of infections had a parasitaemia less than 85.0 parasites/μL. Since current point-of-care RDTs have a threshold of detection of > 100 parasites/μL, they are unlikely to detect the majority of infected infants [31].

In an area coendemic for malaria in Papua New Guinea, where the incidence of malaria was 1700 per 1000 children-years, intermittent preventive treatment with SP and amodiaquine without testing (IPTi) reduced the risk of clinical malaria by 29% and the risk of anaemia by 25% [11]. However, the risk of clinical malaria in our study was 60 fold lower, and in this setting, IPTi is less likely to offer significant clinical benefit disease or be cost effective. Whilst the prevalence of parasitaemia was almost 15% at 12 months, most infections were submicroscopic and asymptomatic. Although the clinical consequences of such low level parasitaemia are unclear, those with subclinical infection were more likely to be anaemic compared to uninfected infants (41% vs 27%), suggesting that interventions to reduce low level infection have potential to prevent anaemia as well as ongoing transmission.

In areas of high malaria endemicity in Africa, maternal and infant malaria intervention strategies are often combined to optimise impact. However, our study in a coendemic high transmission area suggests that the benefits of effective malaria control activities provided during antenatal care may have protected the babies in the first year of life [10, 32–34], potentially accounting for a 10 fold reduction in malaria.

Our study has a number of limitations. First, the study population may have been biased to individuals with a more positive attitude towards malaria, since mothers were attending antenatal care in village health posts, bringing their babies for immunisation and contact with the clinic staff. Additionally, children in the ISTi intervention arm were tested for malaria at 2, 3, 4, 6 and 9 months, increasing the likelihood of detecting a clinical malaria episode compared with the passive detection in the PCDi arm. These potential biases may have reduced the observed effect impact of ISTi. Second, infants in the ISTi arm were more likely to have come from the maternal IST group whereas infants in the PCDi arm were more likely to have been in the maternal IPTp-DHP group. This may have contributed to the reduction in the observed benefit of ISTi since maternal IPTp-DHP was associated with a significant reduction in the risk of malaria during pregnancy compared to maternal IST [13]. Third, the predicted incidence that was used for our sample size calculations was too high and thus our study was underpowered. True incidence data of malaria in the first year of life in this region are scarce, so our estimate was based on experience working in the local hospital where infants can have 2–5 recurrent episodes of malaria within 1 year of their first presentation with malaria. Lastly, almost 20% of patients had incomplete follow-up, with the majority of this attrition bias attributable to migration from the study area, potentially confounding our estimates of ISTi efficacy.

## Conclusions

In an area of high malaria transmission, where *P. falciparum* and *P. vivax* are coendemic, there was no clear benefit to infants receiving ISTi compared to current passive case detection, although this may have reflected reduced power of the study associated with concomitant interventions to reduce maternal malaria. Robust malaria control strategies during pregnancy may provide protection of infants from malaria but not from anaemia. In early life the clinical consequences of submicroscopic asymptomatic infections need to be explored, to inform complementary interventions for mothers and infants at high risk of malaria.

## Supplementary Information

**Additional file 1.**

## Data Availability

The datasets used and/or analysed during the current study are available from the corresponding author on reasonable request.

## Abbreviations

aIRR: Adjusted incidence rate ratio
aPR: Adjusted prevalence ratio
CQ: Chloroquine
DHP: Dihydroartemisinin-piperaquine
DPT: Diphteria, pertussis, tetanus
DSMB: Data Safety Monitoring Board
ICC: Intra-cluster correlation coefficient
IPTi: Intermittent preventive treatment in infants
IPTp: Intermittent preventive treatment in pregnancy
ISTi: Intermittent screening and treatment in infants
ISTp: Intermittent screening and treatment in pregnancy
PCDi: Passive case detection in infants
qPCR: Quantitative polymerase chain reaction
RCT: Randomised controlled trial
RDT: Rapid diagnostic test
SP: Sulfadoxine-pyrimethamine
SSTp: Single screening and treatment in pregnancy
VHP: Village health post
