# Supplementary material for: Intermittent screening and treatment for malaria complementary to routine immunisation in the first year of life in Papua, Indonesia: a cluster randomised superiority trial
Source: BMC Med. 2022 Jun 8;20:190. doi: 10.1186/s12916-022-02394-1 (PMC9175359; doi:10.1186/s12916-022-02394-1)
Supplement: Supplementary file 1 — Additional file 1. [file 12916_2022_2394_MOESM1_ESM.pdf]

Additional file 1

Supplement to: **Intermittent screening and treatment for malaria in the first year of life in Papua, Indonesia: a cluster randomised superiority trial**

Table of contents

1. Infant Malaria laboratory diagnosis by methods 2
2. Infant and Maternal Clusters..3
3. Dosing Regiments...4
4. Statistical Analysis Plan..5
5. Severe Adverse Events 8
6. Maternal Malaria Table..13

1. Infant Malaria laboratory diagnosis by methods

**At 6 Months**	**Microscopy**				
**RDT**	Negative	Pf	Pv	Pf and Pv	**Total**
Negative	661	0	0	0	**661**
Pf	0	5	2	0	**7**
Pf and Pv	0	0	0	0	**0**
**Total**	**661**	**5**	**2**	**0**	**668**

**At 12 Months**	**Microscopy**				
**RDT**	Negative	Pf	Pv	Pf and Pv	**Total**
Negative	609	0	3	0	**612**
Pf	6	6	6	0	**18**
Pf and Pv	0	0	0	0	**0**
**Total**	**615**	**6**	**9**	**0**	**630**

**At 12 Months**	**PCR**				
**RDT**	Negative	Pf	Pv	Pf and Pv	**Total**
Negative	527	55	14	2	**598**
Pf	3	4	3	0	**10**
Pf and Pv	0	0	0	0	**0**
**Total**	**530**	**59**	**17**	**2**	**608**

**At 12 Months**	**PCR**				
**Microscopy**	Negative	Pf	Pv	Pf and Pv	**Total**
Negative	529	55	12	2	**598**
Pf	0	4	0	0	**4**
Pv	0	0	5	0	**5**
Pf and Pv	0	0	0	0	**0**
**Total**	**529**	**59**	**17**	**2**	**607**

2. Infant and Maternal Clusters

Cluster	Village Health Post	Infant's group	Maternal Group	Population	API
1	Kamoro Jaya (SP1)	PCD	IPT	4762	23,1
10	Naena Muktipura (SP6)	IST	IPT	1661	21,67
5	Sempan (Inauga)	PCD	IPT	17868	23,11
20	Karang Senang (SP3)	IST	IPT	3294	35,52
21	Utikini Baru (SP12)	PCD	IPT	1849	35,15
3	Kwamki Baru	IST	IPT	20849	59,8
17	Tipuka	PCD	IPT	277	32,49
6	Wonosari Jaya (SP4)	PCD	IST	3072	23,11
2	Koperapoka	IST	IST	25024	24,5
11	Wangirja (SP9)	PCD	IST	805	22,36
9	Mulia Kencana (SP7)	IST	IST	1441	21,51
16	Poumako	PCD	IST	635	72,44
13	Kadun Jaya	IST	IST	1051	39,96
18	Wania	IST	IST	519	34,68
4	Nawaripi	PCD	SST	1729	23,13
7	Harapan Baru	IST	SST	14147	23,11
8	Mware	PCD	SST	738	21,68
15	Pigapu (Logpong)	IST	SST	335	23,88
14	Kaugapu	PCD	SST	712	40,73
12	Hiripau	IST	SST	817	33,05
19	Bhintuka (SP13)	PCD	SST	1477	42,65

Notes: PCD=Passive Case Detection, IST=Intermittent Screening and Treatment, IPT=Intermittent Preventive Treatment

3. Dosing Regiments

Dihydroartemisinin-Piperaquine (each tablet containing 40 mg dihydroartemisinin and 320 mg piperaquine) will be given orally at 0, 24 and 48 hours.

The target minimum total dose of DHA is 6mg/kg and piperaquine 57mg/kg.

For infants weighing 3 to 17 kg this is given as a solution of 1 tablet crushed and dissolved in 5ml of clean water and administered by syringe.

WEIGHT	DHP SOLUTION	WEIGHT	DHP TABLETS
3 KG	1.0 ML	18 KG	1.5 TABLETS
4 KG	1.3 ML	19 KG	1.5 TABLETS
5 KG	1.5 ML	20 KG	1.5 TABLETS
6 KG	1.8 ML	21 KG	1.5 TABLETS
7 KG	2.1 ML	22 KG	1.5 TABLETS
8 KG	2.4 ML	23 KG	1.5 TABLETS
9 KG	2.7 ML	24 KG	1.5 TABLETS
10 KG	3.0 ML		
11 kg	3.3 ml		
12 kg	3.6 ml		
13 kg	3.9 ml		
14 kg	4.2 ml		
15 kg	4.5 ml		
16 kg	4.8 ml		
17 kg	5.0 ml		

If infants unable to tolerate oral treatment (vomiting) that requires discontinuation of study treatment, intravenous quinine or unsupervised 7 day quinine plus clindamycin will be given. Dose: quinine 10 mg/kg body weight 3 times/day and clindamycin 5 mg/kg body weight 3 times/day for 7 days. This must be recorded in the CRF as **adverse experience(s)** along with the treatment given.

Rescue medication. Patients who fail to respond to the trial drug will be given unsupervised quinine and clindamycin treatment for 7 days (Dose: quinine 10 mg/kg body weight 3 times/day and clindamycin 5 mg/kg body weight 3 times/day). Parasitological and clinical response will be checked at day 3 and 7 after the commencement of oral quinine therapy.

If severe complication(s) occur, intravenous artesunate (2.4 mg/kg bodyweight at 0, 12 and 24 hours) or quinine drips (10 mg/kg bodyweight 8 hourly) must immediately be given along with the required supportive treatments according to the hospital protocol. This will be recorded in a separate severe malaria.
Terminal Eradication. Primaquine **should not** be given to infants.

4. Statistical Analysis Plan

Objectives
To assess the effectiveness of intermittent screening and treatment (ISTi) with dihydroartemisinin-piperaquine (DHP) administered at 2, 3, 4 and 9 months of age compared with the current practice of passive detection and treatment for malaria (PCD).

Research Question
Does intermittent screening and treatment with DHP reduce the incidence of clinical malaria and the prevalence of malaria in the first year of life?

Study population
The study population were infants born to pregnant women enrolled in the STOP MiP trial after the malaria in infancy study started (aged 0-12 months).

Inclusion criteria
Healthy term newborns of consenting mothers living in study areas for the duration of follow-up.

Exclusion criteria
Preterm (<37 weeks gestation) and sick newborns requiring hospitalization will be excluded from the study.

Trial intervention
IST and PCD infants:
Clinical and anthropometry data were available at age 2, 3, 4, 6, 9 and 12 months of age

IST:
Laboratory data (malaria and Hb) were available at age 2, 3, 4, 6, 9 and 12 months of age

PCD:
Laboratory data (malaria and Hb) were available at 6 and 12 months of age and if symptomatic

Endpoints

Primary:
1. Incidence of clinical malaria in the first 12 months of life
2. Prevalence of parasitaemia at 12 months of age

Secondary:
1. Prevalence of anaemia (Hb<10 g/dl) at 6 and 12 months of age

The above endpoints are compared between: IST and PCD group infants (adjusted for maternal intervention)

Definitions

- Clinical malaria: defined as fever or history of fever in the previous 24 hours with asexual peripheral parasitaemia detected by microscopy and/or RDT.
- Parasitaemia: any asexual parasitaemia detected by microscopy, RDT or qPCR.
- Patent parasitaemia: any asexual parasitaemia detected by microscopy and/or RDT
- Subpatent parasitaemia: any asexual parasitaemia not detected by microscopy and/or RDT but detected by qPCR.
- Anaemia: haemoglobin concentration < 10 g/dl
- Maternal parasitaemia: any composite of parasitaemia during pregnancy and at delivery (including placental malaria) diagnosed by any methods (microscopy, RDT, LAMP/PCR) regardless of symptoms.
- Maternal anaemia: haemoglobin concentration < 10g/dl.
- Maternal malnutrition: mid upper arm circumference < 23.5 cm.
- Maternal SES: defined by Principal Component Analysis Methods.

Statistical Analysis

Study flow chart
The flow chart will present the number of infants enrolled and their outcome (loss to follow up, withdrawal consent, died, complete follow up) according to the infant's intervention arms.

Baseline Data and Characteristics
This will be presented by intervention group (IST and PCD). Nominal variables will be presented as N (%) and continuous variables will be presented as mean/median (SD/interquartile range) as appropriate.

Baseline variables include: those of the infant (sex, birth weight, cord haemoglobin concentration, and cord malaria) and mother (age, parity, SES, education, maternal intervention group, maternal parasitaemia at enrolment, during pregnancy and at delivery, maternal haemoglobin level and anaemia at enrolment and delivery and maternal nutritional status for the SST/IST/IPT trial of mothers [Ahmed R et al. Lancet ID 2019]).

Primary outcome analysis
1. Adjusted Incidence Rate Ratio of clinical malaria in infants between IST and PCD group.
The adjusted incidence rate ratio (IRR, 95%CI) of clinical malaria in the first year of life will be compared between IST and PCD group using mixed-effects Poisson regression analysis. Other covariates will be included in the multivariable analysis:

- Maternal (intervention group, parasitaemia at enrolment and during pregnancy, SES (low/medium/high))
- Infant (sex)

2. Adjusted Prevalence Ratios of parasitaemia at 12 months between IST and PCD group

The adjusted prevalence ratios (aPR, 95%CI) of parasitaemia at age 12 months will be compared between IST and PCD group using log binomial regression analysis. Other covariates in the multivariable analysis include:

- Maternal (intervention group, parasitaemia at enrolment or during pregnancy, SES (low/medium/high))
- Infant (sex)

Secondary outcome analysis

1. Adjusted Prevalence Ratios of having anaemia at 6 and 12 months between IST and PCD group

The adjusted prevalence ratios (aPR, 95%CI) of having anaemia at age 6 and 12 months will be compared between IST and PCD group using log binomial regression analysis. Other covariates will be included in the multivariable analysis:

- Maternal (intervention group, parasitaemia at enrolment and during pregnancy, SES (low/medium/high), anaemia and nutritional status)
- Infant (sex)

5. Severe Adverse Events

Table 1. Deaths

No	Study ID	Intervention Arm	Sex	Age (months)	Cause of death	Outcome Notes	Assessment of causality to study drug and study intervention
1	1.16.011	PCD	M	7.2	Severe sepsis due to acute enteritis	Died	Unrelated
2	1.02.033	IST	M	3.3	Heart failure due to severe congenital heart disease (late manifestation) and sepsis	Died	Unrelated
3	1.10.005*	IST	M	8.3	Tuberculous Meningitis, marasmus and severe dehydration	Died	Unrelated
4	1.08.008	PCD	F	12.3	Suspected Child Abuse	Died	Unrelated
5	1.19.014	PCD	M	3.0	Respiratory Failure due to suspected Bronchopneumonia	Died	Unrelated
6	1.12.021#	IST	M	2.5	Died at home: Fever for 7 days (malaria status unknown)	Died	Unrelated
7	1.18.011	IST	F	9.3	Sepsis due to acute enteritis with severe dehydration and Bronchopneumonia	Died	Unrelated
8	1.11.010	PCD	M	7.5	Severe Bronchopneumonia, Cerebral Palsy, Neurodevelopmental Delay	Died	Unrelated
9	1.21.015	PCD	M	9.5	Dyspnoe due to Severe Bronchopneumonia, Metabolic acidosis, Suspected Meningitis DD/ space occupying lesion and septic shock and electrolyte imbalance (secondary hiperpotassemia).	Died	Unrelated

10	1.07.037	IST	M	4.8	Bronchopneumonia DD/ Pertusis with sepsis, Heart failure due to Congenital Heart Disease, Labiopalatoschisis	Died	Unrelated
11	1.16.026	PCD	F	7.7	Acute Watery Diarrhea (dehydration status unknown)	Died	Unrelated
12	1.16.031	PCD	F	11.7	Acute Watery Diarrhea with severe dehydration, Sepsis DD/ Encephalopathy, Hipoglycemia, Acute Kidney Injury	Died	Unrelated
13	1.16.035	PCD	M	8.9	Vomiting with moderate dehydration due to cholestasis jaundice, cardiac and respiratory failure, cerebral herniation, intracranial bleeding due to hemorrhagic disease of the newborn.	Died	Unrelated

Notes: * this infant was admitted two times and left against medical advice, the third admission ended in death. For hospital admissions details please see table 3.[#]the mother did not bring the infant to health facility and died at home.

Table 2. Hospitalization with Malaria

**No**	**Study ID**	**Intervention Arm**	**Sex**	**Age (months)**	**Clinical Assessment**	**Outcome Notes**	**Assessment of causality to study drug and study intervention**
1	1.19.003	PCD	M	2.1	Vivax malaria and anaemia (Hb 6.8 g/dl)	Recovered	Unrelated
2	1.13.003	IST	F	1.5	Vivax malaria and severe anaemia (Hb 4.1 g/dl)	Recovered	Unrelated
3	1.06.026	PCD	F	6.8	Falciparum malaria and hyperpyrexia (t 41^0 C)	Recovered	Unrelated
4	1.21.027	PCD	F	9.7	Malaria Mix, Acute Watery Diarhea moderate dehydration, electrolyte imbalance, oral candidiasis, malnutrition.	Recovered	Unrelated

Table 3. Hospitalization without malaria

No	Study ID	Intervention Arm	Sex	Age (months)	Clinical Assessment	Outcome Notes	Assessment of causality to study drug and study intervention
1	1.16.001	PCD	M	4.5	Sepsis due to acute enteritis	Recovering	Unrelated
2	1.07.001	IST	F	6.6	Measles and Bronchopneumonia	Recovering	Unrelated
3	1.16.008	PCD	F	2.2	Bronchopneumonia	Recovering	Unrelated
4	1.21.003 (1)	PCD	F	4.6	Measles and Bronchopneumonia	Recovering	Unrelated
5	1.21.003 (2)	PCD	F	7.4	Bronchopneumonia, acute enteritis and some dehydration	Recovering	Unrelated
6	1.05.008	PCD	M	6.8	Acute diarrhea with some dehydration	Recovering	Unrelated
7	1.07.009	IST	M	7.8	Acute diarrhea with some dehydration	Recovering	Unrelated
8	1.10.005 (1)	IST	M	6.6	Tuberculous Meningitis, pulmonary TB and marasmus	Leave against medical advice: not recovered	Unrelated
9	1.10.005 (2)	IST	M	7.0	Tuberculous Meningitis, pulmonary TB and marasmus and severe dehydration	Leave against medical advice: not recovered	Unrelated
10	1.08.014	PCD	F	4.8	Bilateral cataracts in infancy	Not recovered	Unrelated
11	1.13.011	IST	M	2.1	Urinary retention due to phymosis and complex febrile convulsion	Recovering	Unrelated
12	1.12.004	IST	M	12.9	Bronchial Asthma severe attack	Recovering	Unrelated
13	1.01.030	PCD	M	4.4	Bronchopneumonia, bronchiolitis and acute watery diarrhea	Recovering	Unrelated
14	1.07.023	IST	F	6.8	Bronchopneumonia	Recovering	Unrelated

15	1.05.042	PCD	M	2.8	Sepsis due to Bronchopneumonia,	Recovering	Unrelated
16	1.07.039	IST	M	4.2	Bronchopneumonia and hypochromic-microcytic anaemia	Recovered	Unrelated
17	1.12.020	IST	M	2.8	Bronchiolitis	Recovered	Unrelated
18	1.16.023	PCD	M	5.6	Bronchopneumonia	Recovered	Unrelated
19	1.16.032	PCD	F	3.7	Bronchopneumonia	Recovered	Unrelated
20	1.18.033	IST	M	4.6	Urosepsis, Cholestasis Jaundice due to Suspected Billiary atresia With Liver failure, Candidiasis Oral	Not recovered	Unrelated
21	1.05.042	PCD	M	3.0	Sepsis due to Bronchopneumonia, Left Pulmonary Atelectasis, hypochromic microcytic anaemia: iron deficiency	Recovering	Unrelated
22	1.04.042	PCD	F	4.5	Sepsis due to diarrhea with moderate dehydration, common cold	Recovered	Unrelated
23	1.07.022	IST	M	6.8	Meningitis tuberculosis (mixed purulent bacterial meningitis) on oral antituberculosis, with developmental disorder, Pneumonia, hypochromic microcytic anaemia	Recovering	Unrelated
24	1.14.016	PCD	F	7.6	Acute diarrhea with mild-moderate dehydration	Recovered	Unrelated
25	1.05.017	PCD	M	12.9	Bronchiolitis	Recovered	Unrelated
26	1.03.024	IST	M	2.1	Bronchopneumonia	Recovered	Unrelated
27	1.21.025	PCD	F	2.0	Constipation due to suspected anal stenosis	Recovering	Unrelated
28	1.03.024	IST	M	4.0	Bronchial asthma, mild attack rare episode, Bronchopneumonia, hypochromic microcytic anaemia due to suspected iron deficiency	Recovering	Unrelated

29	1.02.032	IST	M	4.4	Simple Fibrile seizures due to common cold, Phimosis, Stomatitis	Recovered	Unrelated
30	1.14.007	PCD	M	12.1	Acute watery diarrhea with severe dehydration, common cold	Recovered	Unrelated
31	1.16.032	PCD	F	4.2	Bronchial asthma, rare episode mild attack, Bronchopneumonia dd/Lung TB , Suspected PDA DD/ VSD decompensata, Malnutrition	Recovering	Unrelated
32	1.16.034	PCD	M	4.5	Bronchopneumonia, Hypochromic microcytic anaemia, Malnutrition	Recovering	Unrelated
33	1.18.038	IST	F	6.4	Acute Watery Diarrhea with Common Cold	Recovering	Unrelated
34	1.07.035	IST	M	9.0	Chronic suppurative otitis media auricula dextra et sinistra, Bronchopneumonia, falciparum malaria with hiperparasitemia	Recovered	Unrelated
35	1.06.072	PCD	F	7.3	Urosepsis, Common cold	Recovered	Unrelated
36	1.12.023	IST	F	6.9	Chronic gastroenteritis, electrolyte imbalance, Oral candidiasis, Pneumonia	Recovered	Unrelated
37	1.20.022	IST	F	11.9	Simple Febrile Seizures, acute Gastroenteritis	Recovered	Unrelated
38	1.15.032	IST	M	5.1	Rhinopharyngitis due to bacterial Infection	Recovered	Unrelated
39	1.03.029	IST	F	10.1	Bronchiolitis, suspected sepsis	Recovered	Unrelated

6. Maternal Malaria Table

	PCD	IST	Total
**ANC**			
Peripheral parasitaemia (microscopy) at ANC	57/444 (13%)	42/313 (13%)	99/757 (13%)
Peripheral parasitaemia (RDT) at ANC	25/244 (10%)	12/193 (6.2%)	37/437 (8.5%)
Peripheral parasitaemia (PCR) at ANC	101/444 (23%)	68/313 (22%)	169/757 (22%)
Peripheral parasitaemia (any methods) at ANC	132/444 (30%)	89/313 (28%)	221/757 (29%)
Peripheral Pf parasitaemia (any methods) at ANC	92/444 (21%)	61/313 (19%)	153/757 (20%)
Peripheral Pv parasitaemia (any methods) at ANC	92/444 (21%)	57/313 (18%)	149/757 (20%)
**At delivery (peripheral)**			
Peripheral parasitaemia (microscopy) at delivery	14/340 (4.1%)	8/223 (3.6%)	22/563 (3.9%)
Peripheral parasitaemia (RDT) at delivery	9/400 (2.3%)	6/284 (2.1%)	15/684 (2.2%)
Peripheral parasitaemia (PCR) at delivery	38/400 (9.5%)	24/284 (8.5%)	62/684 (9.1%)
Peripheral parasitaemia (any method) at delivery	46/400 (12%)	29/284 (10%)	75/684 (11%)
Peripheral Pf parasitaemia (any method) at delivery	30/400 (7.5%)	16/283 (5.7%)	46/683 (6.7%)
Peripheral Pv parasitaemia (any method) at delivery	35/399 (8.8%)	18/284 (6.3%)	53/683 (7.8%)
**At delivery (placental)**			
Placental malaria (microscopy) at delivery	14/340 (4.1%)	8/223 (3.6%)	22/563 (3.9%)
Placental malaria (RDT) at delivery	10/380 (2.6%)	7/273 (2.6%)	17/653 (2.6%)
Placental malaria (PCR) at delivery	30/378 (7.9%)	16/270 (5.9%)	46/648 (7.1%)
Placental malaria (any method) at delivery	50/381 (13%)	32/274 (12%)	82/655 (13%)
Placental Pf infection (any method) at delivery	22/399 (5.5%)	11/282 (3.9%)	33/681 (4.8%)
Placental Pv infection (any method) at delivery	31/401 (7.7%)	16/283 (5.7%)	47/684 (6.9%)
Any placental or peripheral malaria infection at delivery	78/401 (19%)	50/285 (18%)	128/686 (19%)
